# Age-Related Decrease in Default-Mode Network Functional Connectivity Is Accelerated in Patients With Major Depressive Disorder

**DOI:** 10.3389/fnagi.2021.809853

**Published:** 2022-01-10

**Authors:** Shixiong Tang, Zhipeng Wu, Hengyi Cao, Xudong Chen, Guowei Wu, Wenjian Tan, Dayi Liu, Jie Yang, Yicheng Long, Zhening Liu

**Affiliations:** ^1^Department of Radiology, The Second Xiangya Hospital, Central South University, Changsha, China; ^2^Clinical Research Center for Medical Imaging in Hunan Province, Changsha, China; ^3^National Clinical Research Center for Mental Disorders, and Department of Psychiatry, The Second Xiangya Hospital, Central South University, Changsha, China; ^4^Center for Psychiatric Neuroscience, Feinstein Institute for Medical Research, Manhasset, NY, United States; ^5^Division of Psychiatry Research, Zucker Hillside Hospital, Glen Oaks, NY, United States

**Keywords:** major depressive disorder, aging, fMRI, functional connectivity, dynamic functional connectivity (dFC), dynamic brain network

## Abstract

Major depressive disorder (MDD) is a common psychiatric disorder which is associated with an accelerated biological aging. However, little is known whether such process would be reflected by a more rapid aging of the brain function. In this study, we tested the hypothesis that MDD would be characterized by accelerated aging of the brain’s default-mode network (DMN) functions. Resting-state functional magnetic resonance imaging data of 971 MDD patients and 902 healthy controls (HCs) was analyzed, which was drawn from a publicly accessible, multicenter dataset in China. Strength of functional connectivity (FC) and temporal variability of dynamic functional connectivity (dFC) within the DMN were calculated. Age-related effects on FC/dFC were estimated by linear regression models with age, diagnosis, and diagnosis-by-age interaction as variables of interest, controlling for sex, education, site, and head motion effects. The regression models revealed (1) a significant main effect of age in the predictions of both FC strength and dFC variability; and (2) a significant main effect of diagnosis and a significant diagnosis-by-age interaction in the prediction of FC strength, which was driven by stronger negative correlation between age and FC strength in MDD patients. Our results suggest that (1) both healthy participants and MDD patients experience decrease in DMN FC strength and increase in DMN dFC variability along age; and (2) age-related decrease in DMN FC strength may occur at a faster rate in MDD patients than in HCs. However, further longitudinal studies are still needed to understand the causation between MDD and accelerated aging of brain.

## Introduction

Major depressive disorder (MDD), one of the most common serious psychiatric disorders worldwide, is associated with increased risks of many biological and physiological pathologies such as dementia/cognitive decline (Byers and Yaffe, 2011; Holmquist et al., 2020), cardiovascular disease (Gan et al., 2014), and osteoporosis (Cizza et al., 2009) which occur in the process of normal aging. Accordingly, there are growing evidences suggesting that MDD leads to an accelerated biological aging as revealed by biochemical (Wolkowitz et al., 2011; Levada and Troyan, 2020), genetic (Simon et al., 2006; Protsenko et al., 2021), and neuroimaging (Sacchet et al., 2017; Cheng et al., 2020; Dunlop et al., 2021) characteristics. For instance, it was found that MDD patients are significantly older (with a median gap of 2 years) than their chronological age based on predictable age-related patterns of DNA methylation (Protsenko et al., 2021). Structural neuroimaging studies have also reported that age-related reductions in the brain cortical thickness (Cheng et al., 2020) and putamen volumes (Sacchet et al., 2017) are accelerated in MDD.

Apart from the above mentioned biochemical, genetic, and brain structural alterations, MDD is characterized by abnormal brain function that can be shown by functional magnetic resonance imaging (fMRI) (Mulders et al., 2015; Zhang K. et al., 2016; Zanatta et al., 2019). In previous fMRI studies, the most prominent and frequently reported findings in MDD are altered resting-state functional connectivity (FC) patterns within the default-mode network (DMN) areas (Mulders et al., 2015; Zhang K. et al., 2016; Zanatta et al., 2019; Shi et al., 2021). Such alterations include both reduced FC strengths (Chen et al., 2015; | Yan et al., 2019; Shi et al., 2020) as well as decreased temporal stability of dynamic functional connectivity (dFC) based on the recent assumption that FC patterns fluctuate over time (Wise et al., 2017; Long et al., 2020a). Interestingly, these alterations have been also associated with the process of normal aging: several previous studies have consistently reported that older age is related to decreased FC strength (Bluhm et al., 2008; Mevel et al., 2013; Vidal-Piñeiro et al., 2014; Park et al., 2017; Staffaroni et al., 2018) and increased dFC variability (Qin et al., 2015; Marusak et al., 2017; Park et al., 2017) within the DMN. Such similarities bring up the possibility that accelerated biological aging in MDD may be reflected by FC strength and dFC variability within the DMN, which has not been well examined to our knowledge. Thus, characterizing the trajectories of age-related changes in FC strength/dFC variability within the DMN may both facilitate the mechanistic understanding of MDD, as well as the development of specific treatment strategies to prevent deteriorated progression in MDD.

In the present study, we therefore investigated whether MDD would be characterized by accelerated aging of brain function in terms of FC/dFC features within the DMN. Specifically, we assessed the MDD diagnosis-by-age interactions on FC strength and dFC variability within the DMN based on the previously mentioned literatures. In order to increase the statistical power and reliability of the results, we used a large, multicenter fMRI dataset of MDD patients and healthy controls (HCs). We hypothesized that (1) age-related decreases in FC strength and increases in temporal variability of dFC within the DMN would be observed in both the MDD and healthy participants; and (2) such processes might be accelerated in MDD as reflected by significant diagnosis-by-age interactions, which are driven by stronger associations between age and FC/dFC measures in patients with MDD.

## Materials and Methods

### Participants

The final analyzed sample in this study consisted of 971 MDD patients and 902 healthy participants from 20 study centers, which was a part of the REST-meta-MDD Consortium in China.^1^ All patients were diagnosed as MDD based on the International Statistical Classification of Diseases, 10th Revision (ICD-10) or Diagnostic and Statistical Manual of Mental Disorders-IV (DSM-IV) criteria. Such a sample was drawn from the original 1300 MDD patients and 1128 HCs in the REST-meta-MDD dataset by excluding the subjects who met the following exclusion criteria: (1) <18 years of age; (2) demographic information such as age is incomplete; (3) fMRI scanning repetition time ≠2 s (to reduce biases caused by different temporal resolutions when constructing dynamic brain networks); (4) poor image quality or inaccurate spatial normalization determined by manual checking; (5) excessive head motion with framewise-displacement (FD) >0.2 mm; (6) bad mask coverage with signal loss in any region of interest (ROI). The data was anonymously contributed from studies which were approved by local Institutional Review Boards in each center, and written informed consent were obtained from all participants from the local institutions.

Among the analyzed 971 patients with MDD, there were 364 first-episode and 234 recurrent MDD patients, while the episodicity (first or recurrent) was unavailable for the other 373 patients. The 17-item Hamilton Depression Rating Scale (HAMD) and Hamilton Anxiety Scale (HAMA) scores were available for 813 and 561 patients, respectively. See Tables 1, 2 for sample details. More details of the REST-meta-MDD Consortium can be also found in previous publications (Yan et al., 2019; Liang et al., 2020; Long et al., 2020a; Ding et al., 2021; Liu et al., 2021; Yang et al., 2021).

**TABLE 1:** The sample size and key data acquisition parameters of each site included in this study.

Site serial number^a^	Samples		Scanner	Repetition time (ms)	Echo time (ms)	Flip angle (°)	Slice number	Time points
	MDD	HCs						
1	71	71	Siemens 3T	2000	30	90	30	210
2	29	26	Philips 3T	2000	30	90	37	200
3	24	33	Siemens 3T	2000	40	90	26	150
6	13	15	Siemens 3T	2000	30	70	33	180
7	32	40	GE 3T	2000	30	90	37	184
8	43	51	GE 3T	2000	30	90	35	200
9	47	48	GE 3T	2000	25	90	35	200
10	28	11	Siemens 3T	2000	30	90	32	240
11	28	27	GE 3T	2000	30	90	33	200
12	31	4	GE 3T	2000	30	90	33	240
15	40	48	Siemens 3T	2000	25	90	36	240
16	28	29	GE 3T	2000	30	90	30	200
17	36	38	GE 3T	2000	40	90	33	240
18	20	18	Philips 3T	2000	35	90	24	200
20	265	241	Siemens 3T	2000	30	90	32	242
21	81	65	Siemens 3T	2000	30	90	33	240
22	22	20	Philips 3T	2000	30	90	36	250
23	27	29	Philips 3T	2000	30	90	38	240
24	24	28	GE 1.5T	2000	40	90	24	160
25	82	60	Siemens 3T	2000	25	90	36	240

*^a^The sample was drawn from data of the original 25 sites in the REST-meta-MDD project. More details about each site (affiliations and principal investigators) can be found at: http://rfmri.org/REST-meta-MDD.*

**TABLE 2:** Comparisons on demographic/characteristics between the MDD and HC groups.

	MDD (*n* = 971), mean ± SD	HCs (*n* = 902), mean ± SD	Group comparisons
Age (years)	37.43 ± 14.80	36.88 ± 16.15	*t* = 0.759, *p* = 0.448
Sex (male/female)	344/627	368/534	χ^2^ = 5.724, *p* = 0.017
Education (years)	11.43 ± 4.09	12.47 ± 4.90	*t* = −4.996, *p* < 0.001
Mean FD (mm)	0.07 ± 0.03	0.07 ± 0.04	*t* = −1.075, *p* = 0.282
Illness duration (months)^a^	38.54 ± 60.64	/	/
HAMD score^a^	20.63 ± 7.83	/	/
HAMA score^a^	19.43 ± 8.90	/	/

*^a^Data on duration of illness, HAMD score and HAMA score was available for 747, 813, and 561 patients, respectively.*

### Imaging Data Acquisition and Preprocessing

All imaging data (including resting-state fMRI and T1-weighted structural images) was required at each center of the REST-meta-MDD Consortium (see Table 1 for key scanning parameters). Data was preprocessed in each center locally with a standardized pipeline to obtain ROI-based fMRI time series. The raw imaging data was not shared to protect participant privacy based on the policy of REST-meta-MDD Consortium (Yan et al., 2019). The preprocessing pipeline was performed using the DPARSF software^2^ (Chao-Gan and Yu-Feng, 2010; Yan et al., 2016) whose details can be found in previous published studies (Yan et al., 2019; Long et al., 2020a). Briefly, it includes removing the first 10 volumes, slice timing, motion realignment, brain tissue segmentation, spatial normalization, temporal filtering (0.01–0.10 Hz), and nuisance (including white matter, cerebrospinal fluid, and whole brain signals) regression. After preprocessing, the images were manually checked by trained researchers to ensure the quality. Data with unsatisfied quality was excluded based on the criteria above in section “Participants.”

### Functional Connectivity Strength and Dynamic Functional Connectivity Variability Within Default-Mode Network

**FIGURE 1:**
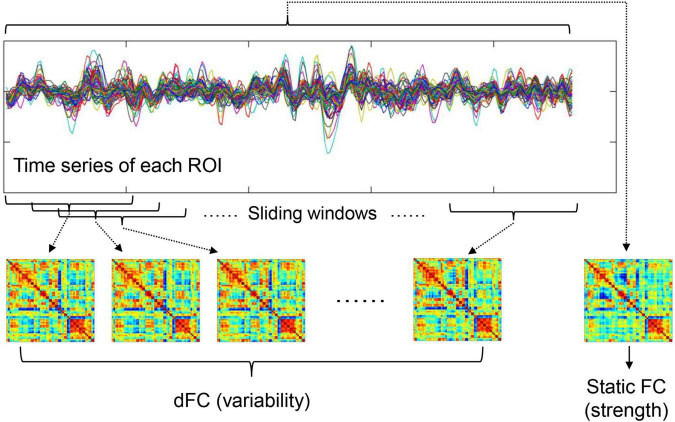
A summary of how to calculate static FC strength and dFC variability (refer to section “Functional Connectivity Strength and Dynamic Functional Connectivity Variability Within Default-Mode Network” for details).

**FIGURE 2:**
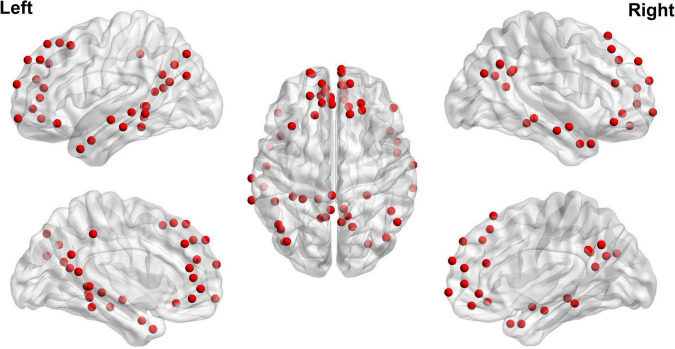
Regions of interest used to define the DMN based on Power et al. (2011).

The steps of calculating DMN FC strength and dFC variability are summarized below and also shown in Figure 1. After preprocessing, mean time series were firstly extracted from 58 DMN ROIs defined based on the Power functional atlas (Power et al., 2011; Cole et al., 2013). The ROIs were visualized in Figure 2 using Brainnet viewer (Xia et al., 2013) and their coordinates can be found elsewhere (Long et al., 2019).

To calculated FC strength within the DMN, the weighted adjacency FC matrices were computed for each participant. In the matrices, FC strengths between all possible (*N* = 58 × 57/2 = 1653 here) ROI pairs were estimated by Pearson’s correlation coefficients between fMRI time series. In line with previous work (Yan et al., 2019), the average FC values between all possible ROI pairs within the DMN was then defined as within-DMN FC strength.

To calculate temporal variability of dFC, a widely used sliding-windows approach (Long et al., 2020a; Huang D. et al., 2021; Huang X. et al., 2021) was applied to segment the time series of all ROIs into a number of continuous time windows; in the primary analyses, a window width of 100 s and a step length of 6 s were used according to previous recommendations (Sun et al., 2019; Long et al., 2020a). The same as static FC, weighted adjacency matrices were computed in each time window to represent dFC during different time periods. Average dFC variability within the DMN was estimated by averaging the dissimilarities of dFC profiles across different time windows. Briefly, node-wise temporal variability of dFC for a ROI *k* (*V*_*k*_) was firstly computed as


$$V_{k}=1-\bar{\text{corrcoef}\left(F\left(i,k\right),F\left(j,k\right)\right)},i,j=1,2,3,\ldots ,T;i\neq j,$$

where *T* is the total number of time windows depending on fMRI scanning length, *F*(*i*, *k*) is the vector characterizing dFC between ROI *k* and all other DMN ROIs within the *i*th time window, and “corrcoef” means correlation coefficients. Temporal variability of dFC for the whole DMN (*V*) was then calculated by averaging *V*_*k*_ of all ROIs within the DMN as


$$\text{V}=\frac{\sum_{\text{k}}{\text{V}}_{\text{k}}}{\text{N}},$$

where *N* is equal to 58 here. More details about such methods can be found in prior studies (Zhang J. et al., 2016; Dong et al., 2019; Long et al., 2020a,b).

### Assessing Diagnosis by Age Effects

Referring to a number of published work (Wright et al., 2014; Sheffield et al., 2016; Sacchet et al., 2017; van Velzen et al., 2020), linear regression analyses were performed to assess the MDD diagnosis by age interactions, with diagnosis (MDD = 0 vs. HCs = 1), age, and diagnosis × age interaction as variables of interest in the prediction of FC/dFC measures, as well as sex, education level, site (as dummy variables), and head motion (mean FD) as covariates (~intercept + diagnosis + age + diagnosis × age + sex+education + site + head motion). Note that here, ‘‘site’’ was included as a covariate to exclude potential effects of differences in fMRI scanning parameters and in the proportions of patients/controls enrolled by each site. Associations between age and FC/dFC measures in each group were further estimated by Pearson correlation coefficients and compared between groups using an online tool, cocor^3^ (Diedenhofen and Musch, 2015), utilizing the function of “comparing correlation coefficients in independent groups” in cocor which is based on procedures provided by Meng et al. (1992) and Zou (2007). All other statistical analyses (except the comparisons between correlation coefficients) were completed in SPSS v22.

### Associations With Clinical Characteristics

Several additional analyses were performed to assess the possible associations between DMN FC/dFC and clinical characteristics. First, to investigate possible associations between the DMN FC/dFC and illness duration/HAMD scores/HAMA scores, partial Pearson correlations were calculated between them in patients whose corresponding information are available, after controlling for age, sex, education, site, and head motion. Second, to investigate if DMN FC/dFC patterns would differ by episodicity (first or recurrent), they were compared between the first-episode and recurrent patients using ANCOVA with covariates of age, sex, education, site, and head motion.

### Validation Analysis

Several supplementary analyses were performed to validate our results. Firstly, in dFC studies, there remain debates about the optimal window width and step length in constructing dynamic brain networks (Qin et al., 2015; Zhang et al., 2018; Savva et al., 2020). Therefore, we repeated the analyses on DMN dFC using a range of different window widths (80/100/120 s) and step lengths (6/8/10 s) to see if the results would be changed. Secondly, we repeated the analyses with Fisher’s *r*-to-*z* transformations on Pearson’s correlation coefficients in all FC/dFC matrices, which were not performed in the main analyses. Thirdly, when dealing with regression analyses, heteroscedasticity is a common problem that may reduce the precision of model (Rosopa et al., 2013). Therefore, we tested the heteroscedasticity of each regression model and when there is a significant heteroscedasticity, we mitigated possible effects of heteroscedasticity by applying a logarithmic transformation to the dependent variable (calculating the natural logarithm) as suggested (Rosopa et al., 2013).

## Results

### Sample Characteristics

Demographic and clinical characteristics of the MDD and HC groups are summarized in Table 2. There were no significant differences in age and head motion between the MDD and HC groups (both *p* > 0.05). The MDD group had a higher proportion of females (χ^2^ = 5.724, *p* = 0.017) and a significantly lower education level (*t* = −4.996, *p* < 0.001) than HCs.

### Linear Regression Results

**FIGURE 3:**
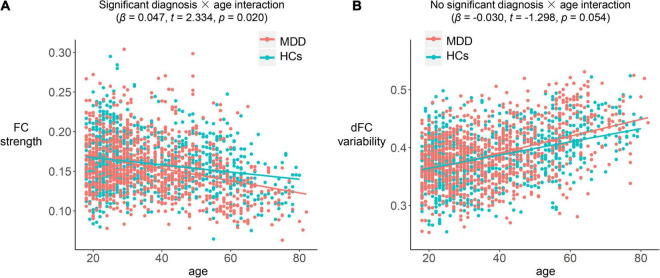
Associations between age and DMN FC strength **(A)**/dFC variability **(B)** in each group.

The linear regression model revealed a significant main effect of age (β = −0.182, *t* = −6.115, *p* < 0.001) in the prediction of DMN FC strength, suggesting that both MDD patients and HCs experienced a similar reduction in DMN FC with age. Moreover, the model revealed a significant main effect of diagnosis (β = 0.074, *t* = 3.631, *p* < 0.001), suggesting a lower DMN FC in MDD patients; and a significant diagnosis × age interaction (β = 0.047, *t* = 2.334, *p* = 0.020) which was driven by a significantly stronger negative correlation (*z* = −2.183, *p* = 0.029) between age and DMN FC strength in MDD patients than in HCs (Table 3 and Figure 3A). Taken together, although the strength of DMN FC decreased with age in both the MDD and HC groups, such reduction may occurr at a faster rate in MDD patients. Additionally, a significant effect of sex (β = 0.061, *t* = 3.030, *p* = 0.002) was found in the model, which suggests a higher DMN FC strength in females than males.

**TABLE 3:** Comparisons on the correlation coefficients between groups.

	In MDD (*n* = 971)	In HCs (*n* = 902)	Group comparisons on *r*
Correlation between age and FC strength	*r* = −0.310	*r* = −0.216	*z* = −2.183, *p* = 0.029
Correlation between age and dFC variability	*r* = 0.434	*r* = 0.394	*z* = 1.043, *p* = 0.297

As for DMN dFC, the linear regression model revealed a significant main effect of age (β = 0.086, *t* = 3.722, *p* < 0.001) in the prediction of dFC variability, suggesting that both MDD patients and HCs experienced a similar increase in DMN dFC variability with age. However, the model revealed no significant main effect of diagnosis (β = −0.017, *t* = −1.046, *p* = 0.296) and no significant diagnosis × age interaction (β = −0.030, *t* = −1.298, *p* = 0.054), with no between-group difference in the correlation between age and DMN dFC variability (*z* = 1.043, *p* = 0.297) (Table 3 and Figure 3B). Additionally, a significant effect of sex (β = −0.047, *t* = −2.894, *p* = 0.003) was found in the model, which suggests a lower DMN dFC variability in females than males.

### Associations With Clinical Characteristics

No significant correlations were found between the DMN FC/dFC and illness duration/HAMD scores/HAMA scores (all *p* > 0.05, Table 4). No significant differences were found between the first-episode and recurrent patients in either the DMN FC strength (*F* = 0.816, *p* = 0.367) or dFC variability (*F* = 1.699, *p* = 0.193).

**TABLE 4:** Correlations between the DMN FC/dFC and clinical characteristics.

	Illness duration	HAMD score	HAMA score
DMN FC strength	*r* = −0.013, *p* = 0.729	*r* = −0.022, *p* = 0.545	*r* = 0.008, *p* = 0.861
DMN dFC variability	*r* = 0.002, *p* = 0.963	*r* = 0.006, *p* = 0.871	*r* = −0.027, *p* = 0.530

### Validation Analysis

When re-calculating the DMN dFC with using a range of different window widths and step lengths, the regression models kept showing a significant main effect of age (*p* < 0.001, see Table 5) in the prediction of DMN dFC variability, while the main effect of diagnosis and diagnosis-by-age interaction kept being no significant (*p* > 0.05). The results on FC and dFC were also largely unchanged when repeating the analyses with Fisher’s *r*-to-*z* transformations on FC/dFC matrices (Supplementary Table 1). These results, therefore, suggest that the effects of age on FC strength/dFC variability were unlikely to be mainly driven by analyzing strategies.

**TABLE 5:** Main effect of age in the prediction of DMN dFC variability, when dFC was calculated with different window widths/step lengths.

Window width (s)	Step length		
	6 s	8 s	10 s
80	β = 0.093, *t* = 3.832 *p* < 0.001	β = 0.094, *t* = 3.851 *p* < 0.001	β = 0.094, *t* = 3.834 *p* < 0.001
100	β = 0.086, *t* = 3.722 *p* < 0.001	β = 0.087, *t* = 3.690 *p* < 0.001	β = 0.087, *t* = 3.662 *p* < 0.001
120	β = 0.079, *t* = 3.660 *p* < 0.001	β = 0.080, *t* = 3.663 *p* < 0.001	β = 0.081, *t* = 3.640 *p* < 0.001

In regression model on the DMN FC strength, there exists a significant heteroscedasticity as indicated by a significant correlation between age and absolute values of the residuals (Spearman’s rho = −0.051, *p* = 0.027; shown in Supplementary Figure 1). Nevertheless, effects of such a heteroscedasticity can be corrected by applying a logarithmic transformation to the DMN FC strength (Supplementary Figure 1), while main results were unchanged in the corrected model (Supplementary Table 2). Therefore, the results were unlikely to be affected by the heteroscedasticity in regression models.

## Discussion

In this study, we tested the hypothesis that MDD would be characterized by accelerated aging of brain function within the DMN as reflected by significant diagnosis-by-age interactions. Specially, age-related effects on both the strength of static FC and temporal variability of dFC within the DMN were investigated by linear regression models. Our results revealed (1) significant main effects of age in the prediction of both FC strength and dFC variability; and (2) a significant main effect of diagnosis and a significant diagnosis-by-age interaction in the prediction of FC strength within the DMN. These results may facilitate our understanding of both the process of biological aging and neural mechanisms underlying MDD.

The first main finding revealed by regression model in the present study is a significant main effect of age in the prediction of both FC strength and dFC variability within the DMN. Such results indicate that both healthy participants and MDD patients experienced similar decrease in DMN FC strength and increase in DMN dFC variability along age. These results are in line with a number of previous studies, which have consistently reported that older age is associated with lower FC strength (Bluhm et al., 2008; Mevel et al., 2013; Vidal-Piñeiro et al., 2014; Park et al., 2017; Staffaroni et al., 2018) and lower dFC stability (higher variability) (Qin et al., 2015; Marusak et al., 2017; Park et al., 2017) within the DMN. It is noteworthy that compared with most of the above studies, our study has a much larger sample size which means a higher reliability (Cao et al., 2019) achieved by using a large, multicenter dataset. Therefore, our results reinforce previous studies and may further offer solid evidence that normal aging of the brain can be reflected by changed FC/dFC patterns within the DMN.

The regression model also revealed a significant main effect of diagnosis, and a significant diagnosis-by-age interaction in the prediction of FC strength within the DMN. The significant main effect of diagnosis indicates a lower DMN FC strength in MDD patients. The DMN is known to mediate brain’s self-referential and internally directed processing (Whitfield-Gabrieli and Ford, 2012) and although not completely consistent (Scalabrini et al., 2020), its FC has been reported to be reduced in MDD in multiple studies (Chen et al., 2015; Jacob et al., 2020; Shi et al., 2020). Thus, our study further supports this result. The significant diagnosis-by-age interaction, which was driven by stronger negative correlation between age and DMN FC in MDD patients (Table 3 and Figure 3A), suggests that age-related reduction in DMN FC may occur at a faster rate in MDD patients than in HCs. Multiple previous neuroimaging studies have reported accelerated brain aging in MDD patients in terms of brain structures (Sacchet et al., 2017; Cheng et al., 2020; Ballester et al., 2021), but much less is known about it from a functional perspective. Here, to our knowledge, our study provides one of the first evidences of accelerated aging of FC within the DMN. Therefore, this brain subsystem may be an important target for the intervention and treatment of MDD across the lifespan.

We should note that in the regression model, no significant main effect of diagnosis and no significant diagnosis-by-age interaction (both *p* > 0.05) were found in the prediction of dFC variability. Thus, the present study failed to replicate previously reported MDD-related increase in DMN dFC variability (Wise et al., 2017; Long et al., 2020a). There are two potential reasons for such inconsistency as we proposed. First, in the analyzed sample we included participants with different fMRI scanning lengths, ranging from 300 to 500 s (Table 1). Although site effects have been controlled in the regression model, such diversity along the time dimension might bring bias to the results. Second, besides the reports of increased DMN dFC variability in MDD (Wise et al., 2017; Long et al., 2020a), there was also research reporting opposite results (Demirtaş et al., 2016). It is possible that subtypes with distinct DMN dFC profiles (hyper- and hypo-variability) may exist in MDD, which can be investigated in future studies.

Although we focused on age-related effects in this study, significant effects of sex were also shown in the regression models, indicating higher FC strength and lower dFC variability within the DMN in females than males. Such results are in concert with some earlier studies, which have pointed out potential sex differences in both static and dynamic brain connectome (Jiang et al., 2020; Long et al., 2021). These results might also, therefore, highlight the necessity of controlling sex-related effects in fMRI studies.

Our study has some limitations. Firstly and importantly, the current study was carried out based on cross-sectional rather than longitudinal data, and we cannot directly define the causation between MDD and accelerated aging. We are also unable to answer whether there are genetic or epigenetic deviations which cause both MDD and more rapid aging. Due to such limitations, it should be only one of possible interpretations of the presented data that MDD causes accelerated aging of brain. Secondly, the detailed clinical information for each MDD patient, such as comorbid conditions, number of prior depressive episodes and treatment details, was not fully recorded due to the variations in data management practices across different sites. This issue has limited our power to further analyze the effects of clinical variables on DMN FC/dFC patterns. Thirdly, because raw image data was not shared by the REST-meta-MDD Consortium, we are unable to further perform voxel-wise analyses (Cui et al., 2018) which may provide valuable information. Lastly, we only focused on FC strength and dFC variability within the DMN based on our literature driven hypothesis. However, other brain subsystems [e.g., salience, affective, and cognitive control networks (Zeng et al., 2012; Manoliu et al., 2014)] and other brain network measures [e.g., static and dynamic small-world metrics (Long et al., 2020a; Yang et al., 2021)] may be also relevant to the occurrence and development of MDD with age, which can be explored in further studies.

In summary, this study tested the hypothesis that MDD is characterized by accelerated aging of the brain function in terms of FC strength and dFC variability within the DMN. Diagnosis-by-age interactions on FC/dFC were estimated by linear regression models. The results suggest that both healthy participants and MDD patients experienced similar decrease in DMN FC strength and increase in DMN dFC variability along age; moreover, stronger negative correlations were found between DMN FC strength and age in MDD patients than in HCs, suggesting that age-related decrease in DMN FC may occur at a faster rate in MDD. These findings may further facilitate our understanding of mechanisms underlying the occurrence and development of MDD. However, further longitudinal studies are still needed to understand the causation between MDD and accelerated aging of brain.

## Data Availability Statement

The datasets presented in this study can be found in online repositories. The names of the repository/repositories and accession number(s) can be found below: http://rfmri.org/REST-meta-MDD.

## Ethics Statement

The studies involving human participants were reviewed and approved by the Ethics Committee of Second Xiangya Hospital, Central South University. The patients/participants provided their written informed consent to participate in this study.

## Author Contributions

ST, ZW, and YL designed the study and carried out the analysis. XC, GW, WT, YL, and ZL contributed to the data collection. ST and YL wrote the first draft of manuscript. All authors contributed to the final manuscript and have read and agreed to the published version of the manuscript.

## Conflict of Interest

The authors declare that the research was conducted in the absence of any commercial or financial relationships that could be construed as a potential conflict of interest.

## Publisher’s Note

All claims expressed in this article are solely those of the authors and do not necessarily represent those of their affiliated organizations, or those of the publisher, the editors and the reviewers. Any product that may be evaluated in this article, or claim that may be made by its manufacturer, is not guaranteed or endorsed by the publisher.

## Footnotes

1
http://rfmri.org/REST-meta-MDD

2
http://rfmri.org/DPARSF

3
http://comparingcorrelations.org/
