# Supplementary material for: Age-Related Decrease in Default-Mode Network Functional Connectivity Is Accelerated in Patients With Major Depressive Disorder
Source: Front Aging Neurosci. 2022 Jan 10;13:809853. doi: 10.3389/fnagi.2021.809853 (PMC8785895; doi:10.3389/fnagi.2021.809853)

**Supplementary Table 1** Results of the regression models when repeating the analyses with Fisher’s r-to-z transformations on FC/dFC matrices.

| Regression model | Main effect of MDD diagnosis | Main effect of Age | Diagnosis × age interaction |
| --- | --- | --- | --- |
| Model on the DMN FC strength | *β* = 0.060, *t* = 2.973, *p* = 0.003 | *β* = -0.221, *t* = -5.985, *p* < 0.001 | *β* = 0.123, *t* = 2.209, *p* = 0.027 |
| Model on the DMN dFC variability | *β* = 0.063, *t* = 1.582, *p* = 0.114 | *β* = 0.144, *t* = 5.107, *p* < 0.001 | *β* = 0.102, *t* = -2.399, *p* = 0.017 |

**Supplementary Table 2** Results of the regression model on DMN FC strength with a logarithmic transformation to the dependent variable (DMN FC strength).

| Regression model | Main effect of MDD diagnosis | Main effect of Age | Diagnosis × age interaction |
| --- | --- | --- | --- |
| Model on the DMN FC strength (with a logarithmic transformation) | *β* = 0.055, *t* = 2.795, *p* = 0.005 | *β* = -0.240, *t* = -6.708, *p* < 0.001 | *β* = 0.169, *t* = 3.132, *p* = 0.002 |

**Supplementary Figure 1** Spearman correlations between the age and absolute values of the residuals in the original regression model on the DMN FC strength (**A**) and the corrected model with a logarithmic transformation to the DMN FC strength (**B**).


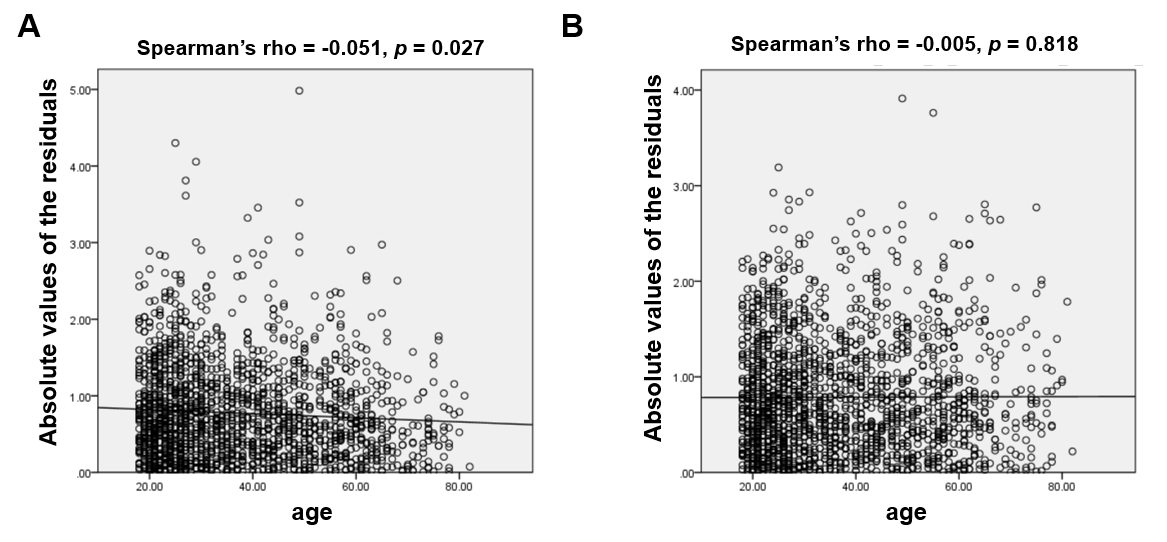

Supplement: Supplementary file 1 [file Data_Sheet_1.docx]
